# Type 2 Diabetes Mellitus and Chronic Heart Failure with Midrange and Preserved Ejection Fraction: A Focus on Serum Biomarkers of Fibrosis

**DOI:** 10.1155/2020/6976153

**Published:** 2020-11-07

**Authors:** D. A. Lebedev, E. A. Lyasnikova, E. Yu Vasilyeva, A. Yu Babenko, E. V. Shlyakhto

**Affiliations:** Almazov National Medical Research Centre, Saint-Petersburg, Russia

## Abstract

As myocardial fibrosis might be an important contributor to the association of diabetes mellitus with left ventricular (LV) dysfunction and chronic heart failure (HF), we investigated the profile of some proinflammatory, profibrotic biomarkers in patients with type 2 diabetes mellitus (T2DM) at various stages of the cardiovascular disease continuum from absence of clinic since and symptoms to HF with preserved (HFpEF) and midrange ejection fraction (HFmrEF). *Material and Methods*. Sixty-two patients with T2DM (age 60 [55; 61]), 20 patients without clinical manifestations of HF and 2 groups with clinical manifestations of stable HF, 29 patients with HFpEF, and 13 patients with HFmrEF, were included in the study. The control group consisted of 13 healthy subjects and normal BMI. All patients underwent transthoracic echocardiography, laboratory assessment of N-terminal fragment of the brain natriuretic peptide (Nt-proBNP), highly sensitive C-reactive protein (hsCRP), soluble suppression of tumorigenesis-2 (sST2), galectin-3, C-terminal propeptide of procollagen type I (PICP), N-terminal propeptide of procollagen type III (PIIINP), matrix metalloproteinase-9 (MMP-9), and tissue inhibitor of matrix proteinase-1 (TIMP-1). *Results*. Patients with HFmrEF had higher values of LV volumetric parameters, indexed parameters of LV myocardial mass (LVMM), and higher concentrations of Nt-proBNP (all *p* < 0.05). The concentrations of galectin-3 were greater in patients with HFpEF and HFmrEF compared to patients without HF (*p* = 0.01 and *p* = 0.03, respectively). PICP and PICP/PIIINP ratio were greater in patients with HFmrEF compared to patients with HFpEF (*p* = 0.043 and *p* = 0.033, respectively). In patients with T2DM and HF, a relationship was found between galectin-3 and LVMM/body surface area (*r* = −0.58, *p* = 0.001), PIIINP, TIMP-1, and LV end-diastolic volume (*r* = −0.68 and *p* = 0.042 and *r* = 0.38 and *p* = 0.02, respectively). *Conclusion*. The dynamics at various stages of the cardiovascular disease continuum in the serum fibrosis markers may reflect an increase in fibrotic and decrease in antifibrotic processes already at the preclinical stage of HF. At the same time, the changes found in the circulating procollagen levels may indicate a shift in balance towards type I collagen synthesis in HFmrEF compared with HFpEF.

## 1. Introduction

Chronic heart failure (HF) and type 2 diabetes mellitus (T2DM) are important problems of most national health systems. Patients with T2DM have a high risk of developing HF, and in a quarter of HF patients with reduced (HFrEF), midrange (HFmrEF), and preserved ejection fraction (EF) (HFpEF) already had T2DM, which significantly worse the prognosis of the disease, increasing mortality by 30-50%. [1, 2].

Taking into account the clinical phenotypic heterogeneity of HFpEF, its pathogenetic mechanisms have not been fully determined. At the same time, the basic characteristics, pathophysiology, and approaches to the treatment of patients with HFmrEF are being actively studied. The pathophysiology of cardiovascular diseases, including HF, in T2DM is multifactorial. T2DM is one of the most significant noncardiac diseases in which a proinflammatory and profibrotic state leads to the development of interstitial myocardial fibrosis, increase in stiffness of myocardium, and a deterioration in elasticity. Also, insulin resistance promotes development of vascular stiffness and hypertension, dyslipidemia, and increased systemic inflammation. Elevated glucose levels lead to increased formation of advanced glycation end-products and accumulation of reactive oxygen species [3]. In combination with visceral obesity, T2DM with a high frequency causes diastolic dysfunction and HF [4]. Hyperglycemia inevitably deteriorates profibrotic processes associated with oxidative, hemodynamic, and metabolic stress in HF [3]. Diabetic cardiomyopathy is usually associated with arterial hypertension (AH) and ischemic heart disease (IHD), forming the so-called “mixed” clinical phenotype of HF, which may be present with both HFpEF and HFmrEF [5]. For specification of pathogenesis of different phenotypes of HF, research of different circulating biomarkers and their diagnostic and prognostic values seem perspective.

Much attention is paid to markers of myocardial stress, such as the N-terminal fragment of brain natriuretic peptide (Nt-proBNP) and markers of inflammation; the most available and studied from which is the highly sensitive C-reactive protein (hsCRP). However, markers associated with profibrotic processes are of particular interest too. Such markers include sST2 (stimulating factor growth expression gene 2, soluble form, also known as IL1RL1, and suppression of tumorigenicity 2), galectin-3, type I procollagen C-terminal propeptide (PICP) and type III procollagen N-terminal propeptide (PIIINP), matrix metalloproteinases (MMP), and their tissue inhibitors (TIMP) [6]. The role of each of these biomarkers is rather specific, and identification of their role in patients with T2DM is complicated by a row of factors. Firstly, T2DM usually occurs in patients with visceral obesity, which is characterized by chronic inflammation and oxidative stress. Thus, it is rather difficult to separate the effects of obesity on biomarker levels from the effects of T2DM itself. Secondly, most of the biomarkers circulating in the blood in HF can be influenced by various factors, including body mass index (BMI) and glycemic levels. Comparison of the serum biomarker levels in patients with similar BMI and similar glycemic control without HF and with HFpEF and HFmrEF will clarify these issues. It should be noted that the relationship between diabetes-related fibrosis and phenotype of HF has not been systematically studied. In addition, little has been studied about difference in patients with T2DM and HFpEF or HFmrEF. In this study, we evaluate fibrosis biomarkers in patients with T2DM and HFpEF or HFmrEF and compare their clinical characteristics and structural and functional parameters of myocardial remodeling.

## 2. Materials and Methods

All procedures were carried out in accordance with the Declaration of Helsinki. The Ethical Committee of the Almazov National Medical Research Centre approved the study (№020419). All patients provided written informed consent. Sixty-two patients with T2DM were included in the study: 20 patients without clinical signs and symptoms of HF (group 1) and 2 groups with clinical manifestations of stable HF: 29 patients with HFpEF (group 2) and 13 patients with HFmrEF (group 3). There was also a control group, which consisted of 13 healthy subjects comparable in age and sex with the studied cohorts, with normal BSA (22-25 kg/m^2^) and heart function. The inclusion criteria in the study were age 40 to 75 years, T2DM, glycated hemoglobin (HbA1c) 7.0-11.0%, and stable hypoglycemic therapy for at least 12 weeks prior to study. In addition, for patients with HF, inclusion criteria were the presence of stable HF (II functional class (NYHA)), HFpEF or HFmrEF, and optimal drug therapy for HF for at least 3 months. The exclusion criteria were valvular heart disease, cardiomyopathy, rheumatological diseases, significant cardiovascular events, including acute myocardial infarction (MI) or major heart surgery, percutaneous coronary intervention (PCI), valvuloplasty within 12 months, and hospitalization due to decompensation of HF within 3 months prior to enrollment in the study; planned intervention on the coronary arteries, HFrEF, III-IV functional class (NYHA), and glomerular filtration rate (GFR) according to Chronic Kidney Disease Epidemiology Collaboration Formula (CKD − EPI) < 60 ml/min/1.73 m^2^; obesity of the III degree; severe bronchial asthma or chronic obstructive pulmonary disease; and secondary arterial hypertension, blood pressure (BP) > 180/110 mmHg, and severe liver disease. The diagnosis of HFpEF and HFmrEF was made according to the recommendations of the European Society of Cardiology 2016 [7]. Laboratory tests were performed, including HbA1c, lipids, and blood creatinine with calculation of GFR, ECG, and echocardiography (VIVID 9 GE, USA) according to a standard protocol by one blinded operator. The left ventricular myocardium mass (LVM) was additionally indexed to the height (in meters). Using height-based indexing (LVM/height in m^2.7^) in individuals with BMI ≥30 kg/m^2^ is preferred in order to avoid underestimation of the prevalence of LV hypertrophy in obese patient [8, 9]. LV hypertrophy was defined using gender-specific criteria with LVM corrected for BSA, height, and height raised to the allometric power of 2.7 according to ASE/EACVI-2015 recommendations on the use of echocardiography in adult hypertension [10]. Intraoperator variability for the assessment of volumetric echocardiographic parameters in our research centre was less than 4%. Longitudinal systolic myocardial deformation was assessed by 2D speckle tracking echocardiography. The automated functional imaging (AFI) software was used for the analysis. The maximum longitudinal strain (GLS) of left ventricle (LV) was calculated by averaging the peak systolic values of 18 segments obtained from 6 segments of three apical views (four-chamber, two-chamber, and long axis of LV). The panel of studied biomarkers included Nt-proBNP, hsCRP, sST2, galectin-3, PICP, PIIINP, MMP-9, and TIMP-1. The serum samples were frozen at -80°C until analysis. The serum hsCRP level was determined on an automatic biochemical analyzer “Cobas Integra 400+” by the immunoturbidimetric method. Serum Nt-proBNP concentration was assessed by electrochemiluminescence method using the Elecsys test system (Roche Diagnostic). Serum levels of galectin-3 (R&D system), MMP-9 and TIMP1 (R&D system), sST2 (Clinical diagnostics, Presage ST2 kit), and PICP and PIIINP (USCN Life Science) were assessed by enzyme immunoassay. Additionally, the ratios of MMP-9/TIMP1 and PICP/PIIINP were calculated for each patient. Echocardiography and blood sampling for biomarkers were performed on the background of sinus rhythm in all patients.

### 2.1. Statistical Analysis

Statistical analysis was performed using the SPSS statistical software (version 21.0, IBM Corp, USA). Continuous variables were presented as medians with 25th to 75th interquartile ranges, and categorical variables were presented as percentages. Continuous variables were compared by Kruskal-Wallis tests followed by Mann–Whitney *U* test when appropriate. Correlations were analysed using Spearman's rank correlation tests. Categorical variables were compared by chi-squared test. Statistical significance was defined as *p* value < 0.05.

## 3. Results

Table 1 shows the main characteristics of the groups. The studied groups had comparable clinical and demographic characteristics. The median age in all groups was about 60 years; the proportion of men was more than 50%. AH was observed in all patients of the studied groups. The duration of AH and the level of office BP did not differ between the groups. Overweight or obesity was diagnosed in all patients. There were no significant differences in BMI and T2DM duration, low-density lipoproteins (LDL), and triglycerides (TG) levels between the groups. HbA1c concentrations in the group without HF, in the group with HFpEF, and in the group with HFmrEF were 8.3%, 8.5%, and 8.8%, respectively (*p* > 0.05). There were no significant gender differences, duration of HF and T2DM, the presence of concomitant diseases, and drug therapy in groups 2 and 3. More than 40% of patients between groups 2 and 3 underwent myocardial revascularization. The incidence of paroxysmal atrial fibrillation among patients with HF varied slightly (15-17%). An endocrinologist and a heart failure specialist consulted all patients with HF. Patients in groups 2 and 3 received the recommended therapy for HF: 77-90% angiotensin-converting enzyme inhibitors (ACEi) or angiotensin receptor antagonists (ARA), 77-90% beta-blockers, 55-61% mineralocorticoid receptor antagonists (MRA), and 100% diuretics. Combined antihyperglycemic therapy in all groups was represented by metformin, sulfonylureas, and/or dipeptidyl peptidase-4 inhibitors (DPP-4).

### 3.1. Echocardiographic Parameters

Echocardiography data are presented in Table 2. Patients with HFmrEF had significantly lower LV EF and lower values of GLS LV compared to patients with HFpEF and patients without HF (*p* 1.2 < 0.05). There was an increase in the left atrial volume index (LA volume index) in patients with HF. This increase was combined with grade 1 diastolic dysfunction in 30% of HF patients. In most cases, patients of all groups had concentric LV myocardial hypertrophy, characterized by an increase in the indexed values of LVM and relative wall thickness (RWT) of the LV. Patients with HFmrEF had significantly higher values of LV end-diastolic volume (EDV) and LV volume index compared to patients with HFpEF and patients without HF (all *p* < 0.05). The indexed parameters of LV MM increased in patients with HF, and their highest values were observed in patients with HFmrEF.

### 3.2. Molecular Biomarkers

Data on the concentrations of biomarkers are presented in Table 3. Lower (50% lower) threshold serum concentrations of Nt-proBNP were used in patients with clinical manifestations of HF, echocardiographic criteria, and BMI > 30 kg/m^2^, taking into account the high incidence of obesity in the study sample [8]. Higher Nt-proBNP concentrations were observed in the group of HFmrEF (*p* < 0.05). The sST-2 concentrations did not significantly differ between the investigated groups and the control group. The concentrations of galectin-3, PICP, PIIINP, and the PICP/PIIINP ratio were significantly higher in all investigated groups compared with the control group (all *p* < 0.05). The concentrations of galectin-3 were greater in patients with HFpEF and HFmrEF compared to patient without HF (*p* = 0.01 and *p* = 0.03, respectively). There was a negative correlation between galectin-3 and high-density lipoprotein (HDL) (*r* = −0.542; *p* = 0.004), which confirmed the participation of this biomarker in the process of atherogenesis. Higher PICP concentrations and the PICP/PIIINP ratio were observed in the group of HFmrEF and in the group without HF compared to the control group. PICP and PICP/PIIINP ratio were greater in patients with HFmrEF compared to patients with HFpEF (*p* = 0.043 and *p* = 0.033, respectively). There was a decrease in the serum concentration of MMP-9 in the groups with HF compared to the control group, although did not quite achieve the threshold for statistical significance. At the same time, the TIMP-1 levels between the studied groups did not significantly differ. The MMP-9/TIMP-1 ratio was significantly lower in all investigated groups compared to the control group (all *p* < 0.05). Furthermore, the decrease in the MMP-9/TIMP-1 ratio was more pronounced in patients with HF. There were no differences in the concentrations of hsCRP, galectin-3, PIIINP, MMP-9, TIMP-1, and the MMP-9/TIMP-1 ratio between patients with HFpEF and HFmrEF. There were no correlations between circulating biomarkers and gender, age, HbA1c, BMI, and drug therapy. There were positive associations between Nt-proBNP concentrations and LA volume index (*r* = 0.44; *p* = 0.003), galectin-3, and values of left ventricular myocardial mass/body surface area (LVM/BSA) (*r* = −0.58, *p* = 0.001), PIIINP and LV EDV (*r* = 0.38; *p* = 0.02), and negative association between TIMP-1 concentrations and LV EDV (*r* = −0.68; *p* = 0.042) in groups with HF.

## 4. Discussion

The list of potential mechanisms contributing to the development of HF in T2DM patients continues to expand each decade. The main processes include oxidative stress, inflammation, impaired sensitivity to insulin, metabolic abnormalities, such as calcium exchange in the myocardium, dysfunction of mitochondria and endothelium, neurohumoral activation, and death of cardiomyocytes. It is not the full list of the difficult processes mediated by hyperglycemia, leading to hypertrophy, excess diffuse extracellular matrix formation, and cardiac steatosis. Cardiac remodeling and impaired microvascular coronary perfusion contribute to the development of diastolic dysfunction [3, 4, 11, 12]. High glucose level stimulates fibroblast proliferation and activates transcription and secretion of extracellular matrix proteins. Hyperglycemia also contributes to increased activity of local renin-angiotensin-aldosterone system (RAAS) in the heart. This increased activity may promote profibrotic processes due to increase in the TGF-*β* expression and reactive oxygen species formation [13].

Most patients with T2DM and HF have comorbidities such as obesity, AH, and accelerated atherosclerosis, which determining the complex clinical phenotype of HF, mainly with LV EF > 40%. The presented study included patients with T2DM, HF with midrange, and preserved LV EF, who have additional cardiometabolic risk factors for HF (increased BMI and AH), and in more than half of the cases, the presence of IHD. It is well known that insulin resistance, metabolic syndrome, and T2DM are associated with an increase in myocardial mass and LV RWT [10] and predispose to the development of concentric LV hypertrophy. Therefore, we included patients with similar BMI and glycemic control. In our study, patients with T2DM and HFmrEF were characterized by increased values of EDV, indexed parameters of LVM, and higher values of Nt-proBNP compared with patients with HFpEF. This observation is consistent with the data of other authors, who conducted a comparative study of echocardiographic parameters in patients with HFpEF and HFmrEF [14]. Similar patterns of echocardiographic changes and Nt-proBNP concentrations were presented in other earlier studies; some authors consider HFmrEF as the initial stage of HFrEF [15]. An increase in LA size is an indicator of a long-term raising in LV filling pressure, and LA volume and function are predictors of an increase in brain natriuretic peptides in patients with HF [14, 16]. We did not find any correlations between the parameters of diastolic dysfunction and the level of Nt-proBNP; however, a strong positive association with the marker of myocardial stress and the LA volume index confirms the association of the LA parameters with neurohormonal activation of the heart in T2DM patients with HFmrEF or HFpEF. Various aspects associated with Nt-proBNP from diagnostics to B-type natriuretic peptide-guided therapy in different populations of patients with HF are actively studied [17–20].

Comparative studies of molecular biomarkers show that patients with HFmrEF have an intermediate biomarker profile associated with myocardial stress, inflammation, and fibrosis [21–23]. Despite the high incidence of T2DM in patients with HFpEF and HFmrEF in these studies, the data on the effect of glycemic status on the molecular biomarkers in these patients are lacking. In our study, we analyzed the wide range of biomarkers in patients with T2DM, multiple cardiometabolic risk factors, and stable HF with LV EF > 40%.

sST2 is a member of the interleukin-1 receptor family and a marker of inflammation and myocardial stress. An increase in the concentrations of sST2 is observed in various cardiovascular diseases, visceral obesity, and T2DM [24]. The significantly lower variability for sST2 in comparison with Nt-proBNP makes it promising for assessing the prognosis of the survival of patients with HF. sST2 is considered as a new marker of cardiovascular events and adverse clinical outcomes, primarily associated with HF and IHD [19]. In our study, serum sST2 in the group with HFpEF did not differ from group with HFmrEF, which is consistent with Song et al. [25]. The lack of differences in serum sST2 concentrations between the control group and study groups can be explained by both the inclusion of patients with mild HF, receiving optimal drug therapy for HF, and the probably low sensitivity of this marker in patients with visceral obesity and T2DM. Despite the prognostic value of sST2 in patients with HFmrEF, its predictive value in patients with concomitant T2DM is doubtful [22–25].

Galectin-3 is a mediator of cell adhesion, inflammation, and extracellular matrix formation both in the myocardium and in the vascular wall [6, 26]. In the studied cohort of patients with T2DM, irrespective of the existence of HF, the increase of galectin-3 was revealed compared to the control group. This observation confirms the intensity of inflammation and fibrosis in patients with T2DM and additional multiple etiological factors of HF, such as AH and obesity, even at the preclinical stage of HF. According to our data, higher serum concentrations of this biomarker and a positive association with LVM in patients with HF confirm the role of galectin-3 in the pathogenesis of HF. However, we did not identify significant differences in galectin-3 concentrations between group with HFpEF and group with HFmrEF. Our data is consistent with another study of patients with HFmrEF and HFpEF; one-third of whom had T2DM and more severe HF [21]. An increase in serum galectin-3 is considered an adaptive response to inflammation and impaired glucose metabolism. However, the data on the predictive value of galectin-3 in relation to other traditional biomarkers in T2DM patients with HF remain ambiguous [24, 27, 28].

According to histological studies, interstitial and perivascular fibrosis in T2DM is associated with an increase in type I collagen and type III collagen, regardless of coronary atherosclerosis and AH [29, 30]. In addition to reactive fibrosis, magnetic resonance imaging often visualizes areas of fibrosis due to asymptomatic coronary events in T2DM patients without MI [31]. These changes contribute to the further remodeling of the myocardium and the progression of HF. The predominant type of the collagen in the extracellular matrix determines by etiological factor of HF. In addition, quantitative and qualitative changes of fibrotic processes are dynamic, presenting different phenotypes of interstitial fibrosis over time, including chronic hyperglycemia [32]. An association between diabetes and an increase in the collagen composition were observed only in patients with HFrEF, according to results of endomyocardial biopsies in patients with T2DM complicated by HFpEF and HFrEF (LV EF < 45%) in the absence of coronary atherosclerosis [29, 33]. Relevant circulating markers of fibrosis include PICP and PIIINP as biomarkers of types I and III collagen formation, respectively [34]. In a number of studies of patients with HF, endomyocardial biopsies have been shown a correlation between the serum PICP concentrations and the total collagen volume in patient with AH, as well as between the PIIINP concentration and the volume of type III collagen in patients with more significant myocardial remodeling in IHD and dilated cardiomyopathy [35–38]. However, there are some controversial issues with these circulating biomarkers in patients with HF [34]. It should be taken into account that in vitro secretion of PICP and PIIINP by fibroblasts is influenced by level of glycemia, AH, and obesity, which complicates their evaluation in patients with T2DM and HF [39–41]. There is a negative association of PIIINP with parameters of subclinical diastolic dysfunction in patients with T2DM in one study [42], while other authors showed a similar association in relation to PICP [43]. There are currently few studies of the described serum markers in HFmrEF, mainly in the meta-analysis of randomized clinical trials in patients with LV EF > 40% [44]. In our study, we identified an increase in the concentrations of PICP and PIIINP in patients with T2DM, regardless of the presence of HF. The PICP levels and the PICIP/PIIINP ratio were significantly higher in the group with HFmrEF compared with the group with HFpEF. PICP/PIIINP ratio was shifted towards the marker of harder collagen in patients with HFmrEF compared to healthy individuals and patients with HFpEF. Furthermore, PIIINP concentration was associated with an increase in LV EDV in all patients with T2DM and HF. The obtained data confirm that a delicate balance between the synthesis and degradation of two types of collagen can determine the structural and functional changes in the myocardium in HF patients with impaired glycemic status. Similar patterns of changes in PICP and PIIINP were observed in the group without HF, which confirms the difficulties discussed above in interpreting the changes of circulating procollagen markers in patients with multiple cardiometabolic risk factors and other possible factors affecting the serum concentrations of these biomarkers. Taking into account the ambiguous data on the diagnostic significance of PICP and PIIINP depending on the etiology of HF, concomitant diseases affecting collagen metabolism, the obtained results of our research require further investigation [34].

Matrix metalloproteinases and their inhibitors play an important role in the exchange of the extracellular matrix, determining the collagen degradation processes. It has been shown that changes in the level of MMP-9, as well as TIMP-1, associated with nonspecific inflammation, and the balance between MMP-9 and TIMP-1 depend on the etiological cause and stage of the cardiovascular disease continuum [26, 34, 45]. Despite numerous evidences of an imbalance in the MMP-9 in T2DM, information about changes in the MMP-9 and TIMP-1 system is contradictory. As in the study by Lewandowski et al., we recorded a decrease in the serum concentration of MMP-9 in patients with T2DM compared with the control group, without reaching the threshold of significance [45]. Low levels of MMP-9 in the studied cohort of patients can be promoted by the drug effects of inhibitors of the renin-angiotensin-aldosterone system, statins, or antihyperglycemic therapy [46–48]. Interestingly, the level of MMP-9 decreased to the greater extent in patients with HF, although not significantly. At the same time, MMP-9/TIMP-1 ratio significantly decreased in all groups of patients compared to the control group, especially in patients with more severe structural and functional heart remodeling. It can indicate the predominance of fibrotic processes in this group of patients. Negative association of TIMP-1 and LV EDV in patients with HF in our study confirms this assumption. The role of matrix metalloproteinases and their tissue inhibitors in the pathophysiology and prognosis in different HF phenotypes is not fully defined and continues to be studied. However, the MMP-9 expression is determined by the nature of the pathological process and the stage of HF [45].

## 5. Conclusion

There is a dynamic balance between the serum concentrations of biomarkers reflecting pro- and antifibrotic processes in patients with T2DM having additional risk factors of HF without and with clinical manifestations of HF in real clinical practice. The dynamics and direction of changes at various stages of the cardiovascular disease continuum in the serum fibrosis markers, MMP-9 and TIMP-1 systems, may reflect an increase in fibrotic and decrease in antifibrotic processes already at the preclinical stage of HF. At the same time, the changes found in the PICP and PIIINP system may indicate a shift in balance towards type I collagen synthesis in HFmrEF compared with HFpEF.

Further prospective studies on large samples using a multibiomarker panel, methods of multivariate statistics in patients with T2DM, and various HF phenotypes are needed.

## 6. Research Limitations

Our study has several limitations. A small sample size as well as the presence of possible confounding factors, including obesity phenotype and other factors not considered in the study, can influence the concentrations of the studied biomarkers of fibrosis. MMP-9 and TIMP-1 were measured in the serum, which can overestimate the concentrations of these biomarkers compared with the levels in plasma samples [43]. The protocol did not include patients with severe HF (III-IV functional class NYHA) and with HFrEF; however, it may be of scientific interest for a more complete understanding of the clinical phenotype of HFmrEF in patients with T2DM.

## Figures and Tables

**Table 1:** Demographic and clinical characteristics of the patients.

Parameters	Group 1 without HF (*n* = 20) Me (25, 75)	Group 2 HFpEF (*n* = 29) Me (25, 75)	Group 3 HFmrEF (*n* = 13) Me (25, 75)	*p* for groups 1, 2, and 3
Age, years	57 (52, 60)	61 (57, 66)	59 (54, 62)	0.44
Gender, male, *n* (%)	13 (65)	15 (51.7)	7 (53.9)	0.74
Duration of CHF, years	—	9 (6, 13)	8 (5, 11)	0.68
CAD, *n* (%)	—	20 (68.9)	11 (84.6)	0.71
MI, *n* (%)	—	15 (51.7)	7 (63.6)	0.85
CABG/PCI, *n* (%)	—	14 (48.2)	7 (63.6)	0.22
Stable angina II, *n* (%)	—	11 (37.9)	6 (46.1)	0.62
Duration of hypertension, years	14 (9, 18)	18 (13, 22)	16 (11; 19)	0.19
SBP, mmHg	130 (115, 140)	140 (110, 155)	130 (110, 145)	0.76
DBP, mmHg	85 (75, 90)	80 (65, 90)	75 (60, 85)	0.62
Heart rate, b/min	77 (68, 90)	70 (62, 78)	68 (63, 74)	0.29
Atrial fibrillation, paroxysmal form, *n* (%)	—	5 (17.2)	2 (15,3)	0.31
Duration of diabetes, years	10 (6, 13)	12 (9, 16)	13 (5, 17)	0.14
BMI, kg/m^2^	32.8 (30.2, 34.9)	32.3 (28.7, 36.8)	34.7 (31.7, 37.8)	0.38
Overweight, *n* (%)	3 (15)	7 (24.1)	2 (15.4)	0.67
Obesity I degree, *n* (%)	12 (60)	17 (58.6)	7 (53.8)	0.9
Obesity II degree, *n* (%)	5 (25)	5 (17.3)	4 (30.8)	0.59
Smoking, *n* (%)	10 (50)	13 (44.8)	6 (46.1)	0.76
Hemoglobin, g/l	144 (128, 157)	140 (125, 159)	138 (120, 151)	0.53
HbA_1c_, %	8.3 (7.7, 9.3)	8.5 (7.9, 9.7)	8.8 (8.4, 9.9)	0.49
GFR, ml/min/1.73 m2,	98 (82, 109)	93 (74, 105)	86 (68, 96)	0.52
LDL, mmol/l	2.7 (2.0, 3.7)	2.1 (1.3, 3.3)	1.9 (1.1, 2.8)	0.65
HDL, mmol/l	1.15 (0.87, 1.28)	1.12 (0.91, 1.35)	0.96 (0.82, 1.33)	0.63
Triglycerides, mmol/l	2.4 (1.8, 3.2)	2.2 (1.7, 2.9)	2.6 (2.1, 3.3)	0.19
ACEi/ARA, *n* (%)	16 (80)	26 (89.6)	10 (76.9)	0.26
Beta-blockers, *n* (%)	—	22 (75.8)	11 (84.6)	0.56
MRA, *n* (%)	—	16 (55.1)	8 (61.5)	0.46
Loop diuretics, *n* (%)	—	19 (65.5)	10 (76.9)	0.28
Thiazide/thiazide-like diuretics, *n* (%)	6 (30)	13 (34.5)	5 (23.1)	0.57
Statins (%)	14 (70)	20 (68.9)	10 (76.9)	0.86
Therapy for DM				
Metformin	19 (95)	29 (100)	13 (100)	—
DPPi-4	14 (70)	18 (62.1)	8 (61.5)	0.82
Sulfonylurea	6 (30)	11 (37.9)	5 (38.5)	

CHF: chronic heart failure; CAD: coronary artery disease; MI: myocardial infarction; CABG: coronary artery bypass grafting; PCI: percutaneous coronary intervention; SBP: systolic blood pressure; DBP: diastolic blood pressure; BMI: body mass index; GFR: glomerular filtration rate; ACEi: angiotensin-converting-enzyme inhibitors; ARA: angiotensin receptor antagonists; MRA: mineralocorticoid receptor antagonists; DPPi-4: dipeptidil peptidase-4 inhibitors.

**Table 2:** Echocardiographic parameters of patients, Me (25, 75).

Parameters	Group 1 without HF (*n* = 20) Me [25; 75]	Group 2 HFpEF (*n* = 29) Me (25, 75)	Group 3 HFmrEF (*n* = 13) Me (25, 75)	*p* value			
				(1 vs. 2 vs. 3)	(1 vs. 2)	(1 vs. 3)	(2 vs. 3)
EF (Simpson), %	61 (59, 65)	61 (57, 62)	46 (41, 48)	<0.001	0.12	<0.001	<0.001
LA dimension, mm	43 (40, 47)	41 (38, 47)	44 (40, 50)	0.29	0.34	0.22	0.21
LA volume index, ml/m^2^	33.5 (30.0, 38.7)	42.0 (37.1, 46.4)	48.3 (38.0, 59.0)	<0.001	0.003	<0.001	0.23
E/e'avg	7 (8, 9)	11 (9, 13)	11 (9, 14)	0.006	0.009	0.005	0.4
PAP, mmHg	26 (19, 30)	24 (18-29)	26 (20-31)	0.57	0.37	0.61	0.18
LV dimension, mm	49 (46, 53)	47 (45, 51)	50 (46, 52)	0.4	0.49	0.43	0.13
EDV, ml	111 (94.7, 126.7)	117 (102, 135)	167 (125, 220)	0.025	0.21	0.005	0.007
LV volume index, ml/m^2^	50 (45, 63)	59 (49, 68)	66 (59, 74)	0.08	0.13	0.03	0.037
ESV, ml	46 (34, 53)	45 (39, 61)	89 (61, 134)	0.007	0.35	0.001	0.002
IVS, mm	10 (9, 11)	11 (10, 13)	11.5 (11, 13)	0.24	0.56	0.39	0.85
PW, mm	11 (10, 12)	11 (10, 13)	11 (9, 12)	0.51	0.43	0.53	0.41
RWT	0.44 (0.41, 0.46)	0.53 (0.49, 0.57)	0.52 (0.5, 0.55)	0.009	0.007	0.01	0.77
LVMM, g	197 (177, 244)	252 (207, 335)	271 (228, 401)	0.01	0.07	0.034	0.28
LVM/BSA, g/m^2^	105 (91, 119)	118 (94, 142)	155 (97, 196)	0.023	0.27	0.02	0.17
LVM/height, g/m	116 (109, 131)	177 (156.0, 192.8)	205.7 (160.1, 220.6)	0.002	0.02	0.001	0.043
LVM/height, g/m^2.7^	49 (42, 56)	52.5 (46.6, 75.1)	69.2 (52.6, 89.3)	0.04	0.15	0.01	0.08
GLS avg, %	-19 (-17, -20)	-19(-17, -20)	-16.5(-14, -19.0)	0.016	0.91	0.041	0.041

EF: ejection fraction; LV: left ventricle; ESV: end-systolic volume of the left ventricle; EDV: end-diastolic volume of the left ventricle; LVM: left ventricular myocardial mass; LA: left atrium; IVS: interventricular septum; PW: posterior wall of left ventricle; RWT: relative wall thickness of the left ventricle; BSA: body surface area.

**Table 3:** Circulating biomarkers in patients and in the control group.

Parameters	Control group (*n* = 13) Me (25, 75)	Group 1 without HF (*n* = 20) Me (25, 75)	Group 2 HFpEF (*n* = 29) Me (25, 75)	Group 3 HFmrEF (*n* = 13) Me (25, 75)	*p* for gr. 1, 2, and 3	*p* for gr. 1 vs. 2	*p* for gr. 1 vs. 3	*p* for gr. 2 vs. 3
Nt-proBNP pg/ml	—	52.4 (14.5, 104.3)	133.6 (78.5, 198.5)	162.5 (134.6, 216.7)	0.005	0.005	0.003	0.03
sST-2, ng/ml	18.3 (15.6, 21.2)	19.2 (16.2, 26.3)	19.9 (12.9, 40.7)	19.6 (13.8, 37.1)	0.97	0.89	0.77	0.28
Galectin-3, ng/ml	5.7^+^ (4.9, 6.3)	8.6 (7.2, 10.8)	11.7 (8.1, 13.4)	10.4 (7.9, 14.0)	0.03	0.01	0.03	0.84
PICP, ng/ml	15.0^+^ (10.0, 34.0)	127.4 (113.3, 170.2)	46.8 (22.6, 98.6)	106.4 (85.4, 140.4)	0.005	0.006	0.09	0.043
PIIINP, ng/ml	1.27^+^ (0.67, 3.18)	3.38 (2.85, 3.61)	3.29 (1.96, 4.59)	3.46 (2.24, 4.97)	0.58	0.68	0.49	0.38
PICP/PIIINP	7.4^+^ (3.7, 8.4)	35.3 (30.1, 44.4)	18.4 (14.6, 25.3)	31.5 (25.8, 40.1)	0.07	0.017	0.35	0.033
MMP-9, ng/ml	540.0 (363.0, 669.0)	412.5 (289.5, 687.7)	277.0# (98.5, 403.5)	257.0 # (152.0, 483.5)	0.12	0.26	0.11	0.51
TIMP-1, ng/ml	149.0 (133.0, 170.0)	208 (172, 241)	178.5 (119.5, 210.7)	154 (114.7, 203.5)	0.16	0.21	0.05	0.81
MMP-9/TIMP-1	3.6^+^ (2.3, 5.2)	1.6 (1.2, 2.8)	1.1 (0.6, 3.6)	0.8 (0.42, 3.1)	0.31	0.44	0.39	0.5
hsCRP, mg/l	—	2.1 (0.8, 4.9)	2.5 (1.7, 5.9)	3.3 (2.0, 7.3)	0.41	0.29	0.31	0.61

^+^ *р* < 0.05 (between control group and investigated groups); #*р* < 0.03 (between control group and groups 2 and 3).

## Data Availability

Research data can be available after contact by e-mail doctorlebedev11@gmail.com with corresponding author.
